# Detection of virulence factors of South African *Lactococcus garvieae* isolated from rainbow trout, *Oncorhynchus mykiss* (Walbaum)

**DOI:** 10.4102/ojvr.v85i1.1568

**Published:** 2018-10-04

**Authors:** Cornelia M. Meyburgh, Robert R. Bragg, Charlotte E. Boucher

**Affiliations:** 1 Department of Microbial, Biochemical and Food Biotechnology, University of the Free State, South Africa

## Abstract

*Lactococcus garvieae* is a Gram-positive bacterium that causes mortalities in freshwater and marine fish worldwide and therefore results in severe economic losses in the aquaculture industry. Apart from the apparent integral role of the exopolysaccharide (EPS) capsule in pathogenesis, factors associated with virulence of this bacterium are poorly understood. However, recent studies have indicated that the ability of *L. garvieae* to cause disease does not depend on the presence of the EPS capsule. Lack of knowledge of virulence factors, pathogenesis and serology of *L. garvieae* is an impediment to the development of effective typing methods and control measures. This study, therefore, aimed to detect the presence of EPS capsules and other putative virulence factors in South African *L. garvieae* fish pathogenic isolates and a non-virulent isolate, and to identify possible candidates for subunit vaccine development. No indication of the presence of the EPS capsule was detected by negative staining or amplification of the EPS biosynthesis gene cluster in the virulent isolates or the avirulent strain, discrediting the notion that the EPS capsule is the sole determinant of virulence. However, a set of putative virulence factor genes was detected in all isolates, and candidates for subunit vaccine development (enolase, lactate dehydrogenase phosphoenolpyruvate-protein phosphotransferase) were identified by identification of extracellular proteins of virulent strains.

## Introduction

Infectious disease caused by bacteria causes severe fiscal loss in aquaculture (Austin & Austin 2012). *Lactococcus garvieae,* a pathogen of importance in the aquaculture of freshwater and marine fish (Bragg & Broere 1986; Collins et al. 1983; Eldar et al. 1996; Kusuda et al. 1991), is described as a Gram-positive, facultative anaerobic, non-motile bacterium that does not produce endospores. It is generally described as an α-haemolytic bacterium (Ravelo et al. 2001), but has been noted as *β*-haemolytic (Teixeira et al. 1996). A South African Gram-positive cocci isolate, initially described as *Streptococcus* spp. (Bragg & Broere 1986), were reclassified as *Enterococcus* spp. and *L. garvieae* based on 16S ribosomal deoxyribonucleic acid DNA (rDNA) sequencing (Bekker et al. 2011). The bacterium is considered an emerging zoonotic agent, placing immunocompromised individuals at risk (Gibello et al. 2016).

Antimicrobial agents show strong *in vitro* activity against *L. garvieae,* but perform poorly under field conditions because of anorexia of infected fish (Bercovier et al. 1997) and possibly the ineffective metabolism of antibiotics in fish (Romero, Feijoó & Navarrete 2012). Dissemination of antibiotic resistance in bacteria has grown into a global public health concern, accelerated by the unregulated and injudicious administration of antibiotics in humans and animals (Heuer et al. 2009). In aquaculture, chemotherapeutic treatment has led to the emergence of multiple resistance in streptococcal fish pathogens (Aoki et al. 1990; Austin & Austin 2012). The spread of antibiotic resistance is aided by various mechanisms of horizontal gene transfer, of which plasmid-mediated transfer is the most widely documented in streptococcal fish pathogens. Vaccination is considered the best option to control lactococcosis because of the poor efficiency of chemotherapeutic agents under field conditions and the risks associated with the spread of antibiotic resistance determinants.

Bacteria employ a wide repertoire of virulence factors to promote survival within the host, some of which cause imminent damage to the host. Early studies on toxins of a non-Lancefield *Streptococcus* sp. (presumably *L. garvieae*) isolated from yellowtail showed the presence of a haemolytic toxin in culture supernatant (Kusuda & Hamaguchi 1988). A study by Aguado-Urda et al. (2012) characterised five plasmids in a clinical isolate of *L. garvieae* strain 21881. The largest of these plasmids (68 798 bp [base pair]), pGL5, was shown to encode putative virulence factors, including a protein that posesses the enzymatic domain corresponding to the family of actin-ADP-ribosyltransferases (Aguado-Urda et al. 2012). Full genome sequencing of *L. garvieae* strains ATCC 49156^®^ and Lg2 by Morita et al. (2011) identified several genes encoding putative virulence factors showing significant similarity to virulence factors of related species.

A comparative genome analysis of a virulent strain Lg2 and a non-virulent strain ATCC^®^ 49156 of *L. garvieae* identified a 16.5 kilobase (kb) capsule gene cluster, which is present in Lg2 but absent in ATCC^®^ 49156 (Morita et al. 2011). The capsular gene cluster consists of 15 genes, of which 8 (*eps-R, X, A, B, C, D* and *cps-L, W*) are conserved in the exopolysaccharide (EPS) biosynthesis gene cluster of four *Lactococcus lactis* strains isolated from human faecal samples (Morita et al. 2011). Analyses indicate that the capsular gene cluster is a genomic island, because of the presence of insertion sequences (ISs) on both ends of the capsular gene cluster. Even though the polysaccharide capsule is widely regarded as a major virulence factor of *L. garvieae,* it has been shown that non-capsulated strains Lgper and ATCC^®^ 49156 are pathogenic to rainbow trout, causing 89% and 98% mortality, respectively (Türe et al. 2014). These results suggest that the presence of the polysaccharide capsule cannot be directly correlated with pathogenicity in fish.

Despite the integral role of the polysaccharide capsule in pathogenesis, none has been structurally characterised. Understanding of the underlying genetic basis of variability in EPS structure and its likely interrelationship with serological variability in *L. garvieae* is lacking presently. Lack of knowledge of virulence factors, pathogenesis and serology of *L. garvieae* is an impediment to the development of effective typing methods and control measures. Therefore, this study aims to investigate the prevalence of various putative virulence factors in South African *L. garvieae* isolates and to speculate on their role in the process of infection in order to further elucidate the pathogenesis of lactococcosis in rainbow trout and identify possible targets for recombinant vaccine development.

## Materials and methods

### Isolates used in this study

Cultures used in this study were obtained from a study by Bragg and Broere (1986) during which these bacteria were isolated from diseased rainbow trout, *Oncorhynchus mykiss* (Walbaum), in the former Eastern Transvaal (currently Mpumalanga) area and Johannesburg, Gauteng. Symptoms observed in diseased rainbow trout included extreme exophthalmos and rupture of one or both eyes, haemorrhaging of the ocular chamber, melanosis, enlargement of the spleen and haemorrhaging of intestines (Bragg & Broere 1986). Geographic origins and isolate numbers (A1–A12), which will hereafter be used to refer to isolates, are presented in Table 1. Freeze-dried isolates were revived in brain heart infusion (BHI) broth (Merck, 1.10493) and incubated under aerobic conditions at 30 °C (A1–A3, A5, A6, A11 and A12) for 48 hours or anaerobic conditions at 37 °C (A4, A7–A10) for 48 h using an anaerobic jar with a gas-generating kit (Oxoid™ BR0038, Basingstoke, United Kingdom). Culture purity was confirmed by Gram staining. Revived isolates were stored in commercially available cryogenic vials (Microbank™, Pro-Lab Diagnostics, Toronto, Canada) at -20 °C.

**TABLE 1:** Isolate numbers and geographic origins of isolates used in the current study.

UFS number	Isolate number	Geographic origin
UFSBC574	A1	TPA Lydenburg
UFSBC575	A2	Farm near Lydenburg
UFSBC576	A3	Farm near Lydenburg
UFSBC577	A4	Maloney’s Eye – Johannesburg
UFSBC578	A5	G. Coetzee – Lydenburg
UFSBC579	A6	Lunsklip Fisheries – Lydenburg
UFSBC580	A7	Exact location unknown
UFSBC581	A8	Exact location unknown
UFSBC582	A9	Exact location unknown
UFSBC583	A10	Exact location unknown
UFSBC548	A11	Freshwater crab – Maloney’s Eye
UFSBC547	A12	Dullstroom – Pleasant Ways trout farm

UFS, University of the Free State; TPA, Transvaal Provincial Administration.

### Identification of isolates by 16S ribosomal DNA sequencing

Isolates were cultivated in tryptic soy broth (TSB) (Merck, 1.05459) under aerobic conditions at either 30 °C or 37 °C for 18 h with no agitation. DNA was extracted according to the method described by Thanh (2006). Universal sequence primers 8F (5’-AGAGTTTGATCCTGGCTCAG-3’) and 1525R (5’-AAGGAGGTGATCCAGCC-3’) were used to amplify a 1517 base pair (bp) sequence of the 16S rDNA gene by polymerase chain reaction (PCR) (Beumer & Robinson 2005). Amplicons were visualised by gel electrophoresis on a 1% agarose gel (Sambrook & Russell 2001). For purposes of purification of the PCR product, the PCR products were run on a 2% weight per volume (w/v) low-melt agarose (NuSieve^®^ GTG^®^ Agarose). Bands were excised under ultraviolet illumination. The PCR products were purified from gel slices using a Wizard^®^ SV Gel and PCR Clean-up System (Promega, Madison, WI). Sequencing reactions were prepared using Applied Biosystems™ BigDye^®^ terminator v.3.1 sequencing kit. Samples were submitted for Sanger sequencing at an in-house facility at the University of the Free State. Sequence data were viewed using Geneious version 9 (http://www.geneious.com, Kearse et al. 2012) and sequences were used to query the National Centre for Biotechnology Information (NCBI) database using the nucleotide–nucleotide BLAST (BLASTn) algorithm (Altschul et al. 1990).

### Phenotypic characterisation of exopolysaccharides

Negative staining with nigrosine was performed on isolates A1–A12 to determine the presence of EPS capsules. *Pseudomonas aeruginosa* and an avirulent reference strain of *L. garvieae* (NCFB657) were included as positive and negative controls, respectively.

### Genotypic characterisation of exopolysaccharides

The *L. garvieae* EPS synthesis gene cluster, as described in *L. garvieae* Lg2 (Miyauchi et al. 2012), was amplified in isolates A1–A3, A5, A6, A11 and A12 using the TaKaRa LA PCR Kit (TaKaRa Bio Inc. #RR002A, Kyoto, Japan). Amplicons were visualised on a 1% agarose gel.

### Detection of putative virulence factor genes by polymerase chain reaction

The presence of putative virulence factor genes, as identified by Morita et al. (2011), in South African isolates were investigated by PCR using primers designed by Türe and Altinok (2016). The presence of the following genes was investigated: haemolysins 1, 2 and 3 (*hly*1, -2, -3), NADH oxidase (*nox*), superoxide dismutase (*sod*), pneumococcal adherence and virulence factor A (*pavA*) and pneumococcal surface adhesin A (*psaA*) (Table 2). Amplicons were visualised on a 1% agarose gel. Products were purified using a Wizard^®^ SV Gel and PCR Clean-up System (Promega, USA). Sequencing was performed using the Applied Biosystems^™^ BigDye^®^ terminator v.3.1 sequencing kit. Obtained sequences were viewed and edited with Geneious 9 (Kearse et al. 2012) and used to query the NCBI database using the BLASTn algorithm (Altschul et al. 1990).

**TABLE 2:** Genetic loci and predicted gene products of candidate virulence genes, previously identified in *Lactococcus garvieae* Lg2 (Morita et al. 2011), under investigation in this study.

Locus	Predicted gene product	Identity (%)	Species	Accession number	Features
LCGL_0323	Haemolysin	56	*Enterococcus faecalis*	AAO81463	Motif (PF03006) conserved in proteins with haemolytic activity
LCGL_0597	Haemolysin	72	*Streptococcus suis*	EEF64743	-
LCGL_0374	Haemolysin	59	*S. pyogenes*	AAK33420	Cleavable N-terminal signal sequence
LCGL_0664	NADH oxidase (SP1469)	51	*S. pneumoniae TIGR4*	-	-
LCGL_0285	Superoxide dismutase (SP0766)	68	*S. pneumoniae TIGR4*	-	-
LCGL_1330	PavA (SP0966)	62	*S. pneumoniae TIGR4*	-	Fibronectin-binding motif (PF05833)
LCGL_1533	PsaA (SP1650)	49	*S. pneumoniae TIGR4*	-	-

*Source*: Bekker, A., Hugo, C., Albertyn, J., Boucher, C.E. & Bragg, R.R., 2011, ‘Pathogenic Gram-positive cocci in South African rainbow trout, *Oncorhynchus mykiss* (Walbaum)’, *Journal of Fish Diseases* 34(6), 483–487. https://doi.org/10.1111/j.1365-2761.2011.01259.x

### Detection of extracellular virulence factors

*Lactococcus garvieae* strains A1–A3, A5, A6, A11, A12 and NCFB657 were cultivated in TSB media for 24 h at room temperature. A negative control, consisting of sterile TSB, was included. Cells were pelleted by centrifugation at 20 000 x *g* for 2 minutes, and the supernatants filtered with a 0.2 *μ*m syringe filter unit to remove residual cellular components. Amicon^®^ Ultra-4 10K Centrifugal Filter Units (Merck) with a molecular weight cut-off (MWCO) of 10 kDa were used to concentrate extracellular proteins. A volume of 4 mL filtered supernatant was applied to the centrifugal filter and the unit was centrifuged at 7197 x *g* for 20 min. The eluate was discarded, the centrifugal filter was inverted inside a 50 mL Falcon tube and again centrifuged at 7197 x *g* for 20 min to collect the concentrated protein fraction. Extracellular proteins, concentrated by Amicon^®^ Ultra-4 10K Centrifugal Filter Units (Merck), were visualised by sodium dodecyl sulphate-polyacrylamide gel electrophoresis (SDS-PAGE). The polyacrylamide gel was stained and destained using the rapid Fairbanks Coomassie Blue protein staining and destaining method (Wong et al. 2000).

#### Protein identification

Prepared samples were submitted to the Facility for Genomics and Proteomics at the University of the Free State for identification by nanoscale liquid chromatography coupled to tandem MS (Nano LC-MS/MS) and by querying the SWISS-PROT annotated protein sequence database (Bateman et al. 2015) using the Mascot search engine that uses a probability-based scoring algorithm based on the molecular weight search (MOWSE) algorithm (Perkins et al. 1999).

## Results

### Identification of isolates

**FIGURE 1:**
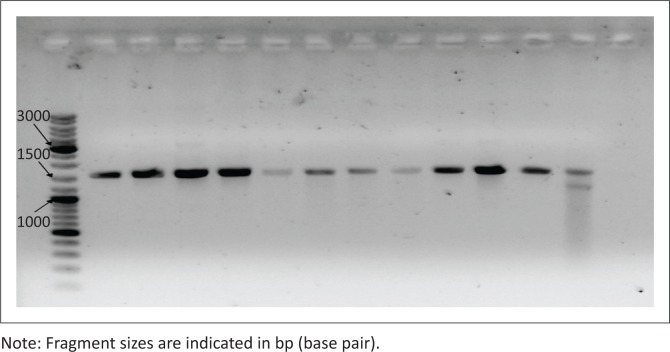
Visualisation of 16S polymerase chain reaction products of isolates A1–A12 on a 1% weight per volume (w/v) agarose gel. Bands in the expected size range (± 1500 bp) were observed.

Amplification of the 16S rDNA gene fragment by PCR yielded the expected ± 1500 bp product, visualised by gel electrophoresis in Figure 1. The nucleotide BLAST search results, tabulated in Table 3, identified isolates A1–A3, A6, A11 and A12 as *L. garvieae,* A4 as *Enterococcus faecalis*, A7 as *E. durans* and A8–A10 as *E. faecium*. These results are consistent with the results of Bekker et al. (2011), who also identified this set of 12 isolates as 5 *Enterococcus* spp. and 7 *L. garvieae* isolates using 16S rDNA sequencing (Bekker et al. 2011). No reports of fish-pathogenic *Enterococcus* spp. have been published; therefore, isolates A4 and A7–A10 were excluded from further experiments in this study.

**TABLE 3:** Top Basic Local Alignment Search Tool search hits generated for 16S ribosomal DNA genes amplified from isolates A1–A12.

Isolate	Nucleotide BLAST result	Query length	Query cover (%)	E value	Identity (%)	Accession nr
A1	*Lactococcus garvieae* strain A4 16S ribosomal RNA gene, partial sequence	619	100	0.0	100	KT924257.1
A2	*Lactococcus garvieae* strain A2 16S ribosomal RNA gene, partial sequence	783	100	0.0	100	KT924256.1
A3	*Lactococcus garvieae* strain RCB99 16S ribosomal RNA gene, partial sequence	742	100	0.0	100	KT260311.1
A4	*Enterococcus faecalis* strain RCB986 16S ribosomal RNA gene, partial sequence	466	100	0.0	99	KT261198.1
A5	*Lactococcus* sp. SI-Km(R)-3B 16S ribosomal RNA gene, partial sequence	101	100	2e-39	97	KR399992.1
A6	*Lactococcus garvieae* strain RCB131 16S ribosomal RNA gene, partial sequence	80	100	8e-33	100	KT260343.1
A7	*Enterococcus durans* strain R08-28 16S ribosomal RNA gene, partial sequence	44	100	1e-14	100	JF896442.1
A8	*Enterococcus faecium* strain 133 16S ribosomal RNA gene, partial sequence	166	98	5e-54	91	EU418442.1
A9	*Enterococcus faecium* strain SA-10 16S ribosomal RNA gene, partial sequence	574	100	0.0	100	KR265372.1
A10	*Enterococcus faecium* strain UW7606x64/3 TC1, complete genome	354	100	0.0	99	CP013009.1
A11	*Lactococcus garvieae* strain RCB99 16S ribosomal RNA gene, partial sequenc	405	100	0.0	100	KT260311.1
A12	*Lactococcus garvieae* strain RCB130 16S ribosomal RNA gene, partial sequence	96	98	5e-31	94	KT260343.1

BLAST, Basic Local Alignment Search Tool; RNA, ribosomal nucleic acid.

### Phenotypic characterisation of exopolysaccharides

#### Negative staining

**FIGURE 2:**
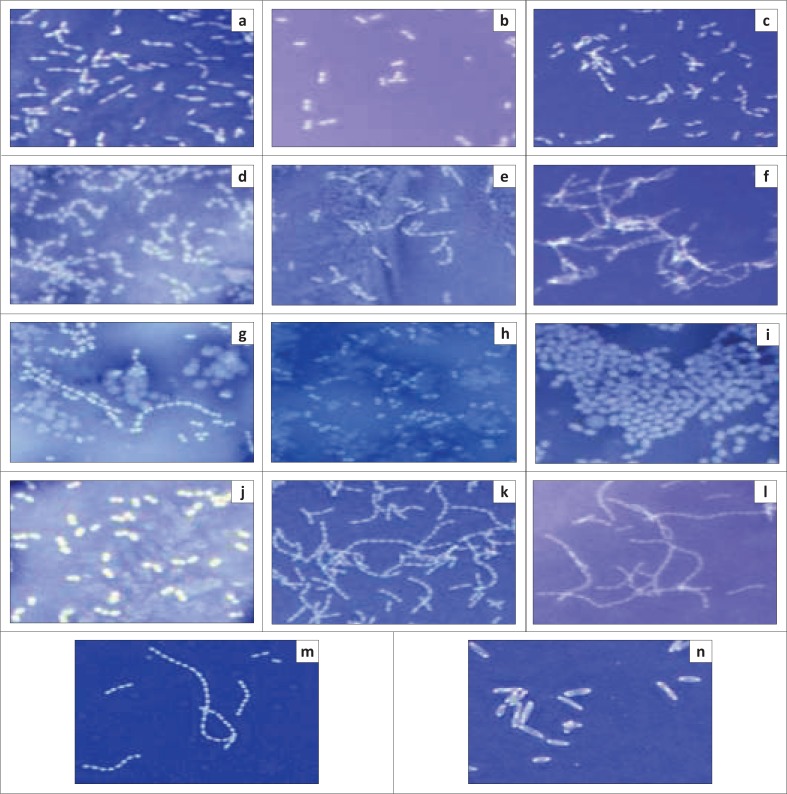
**N**egative staining of *Lactococcus garvieae* A1–A12 using nigrosin, visualised using 100x magnification. *L. garvieae* NCFB657 and *Pseudomonas aeruginosa* were included as negative and positive controls, respectively. (a), A1; (b), A2; (c), A3, (d), A4; (e), A5; (f), A6; (g), A7; (h), A8; (i), A9; (j), A10; (k), A11; (l), A12; (m), *L. garvieae* NCFB657; (n), *P. aeruginosa*.

As shown in Figure 2, no halos were observed in any of the isolates including the negative control NCFB657, in comparison with the positive control *P. aeruginosa*, where a clear halo was observed, indicating the presence of a polysaccharide capsule.

### Genotypic characterisation of exopolysaccharides

**FIGURE 3:**
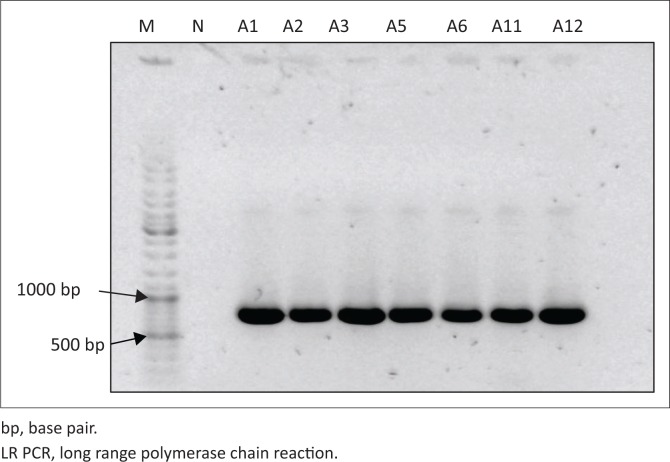
Visualisation of LR PCR products on a 1% agarose gel. A band of ~ 750 bp was observed in all isolates tested, indicating the absence of an exopolysaccharide gene cluster in the genomes of these isolates.

The amplification of the EPS biosynthesis gene cluster by long-range PCR was attempted as described by Miyauchi et al. (2012). Figure 3 shows a product of ~ 750 bp for all isolates tested, consistent with the product sizes observed in the KG^+^ (non-capsulated) strains tested in the study by Miyauchi et al. (2012). The presence of insertion sequences (ISs) flanking the EPS biosynthesis gene cluster in the KG^−^ strain Lg2 used in the study by Miyauchi et al. (2012) may suggest that the EPS biosynthesis gene cluster could be inserted at different genomic loci in strains other than Lg2. If this should happen, the primers used by Miyauchi et al. (2012) will fail to amplify this gene cluster in other strains as these primers flank the gene cluster including the ISs. Therefore, primers that amplify the first gene in the cluster, a transcriptional regulator (*epsR*), were designed. However, PCR amplification of this gene yielded no product in any isolates tested.

### Detection of extracellular proteins

**FIGURE 4:**
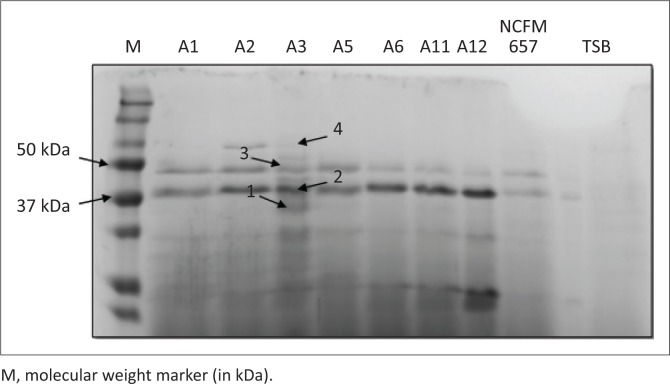
Extracellular proteins of eight *Lactococcus garvieae* strains, including the reference strain NCFB657, visualised on a 12% sodium dodecyl sulphate-polyacrylamide gel electrophoresis gel. A negative control (sterile tryptic soy broth (TSB)) was included.

Extracellular proteins of eight *L. garvieae* strains, purified and concentrated by Amicon^®^ Ultra-4 10K Centrifugal Filter Units (Merck), were visualised on a 12% SDS-PAGE gel. More protein bands were observed in strain A3 in comparison to other strains, as can be seen in Figure 4. No clear bands were observed in the negative control, which consisted of sterile TSB medium, indicating that the growth medium did not contribute any of the protein bands detected and identified in supernatants (Table 4).

**TABLE 4:** Top protein scores generated by query of the SWISS-PROT protein sequence database with the search engine Mascot, following nano liquid chromatography–tandem mass spectrometry analysis of extracellular proteins from *Lactococcus garvieae* strain A3.

Band no.	Top protein scores	MOWSE score (*p* < 0.05)	MW (kDa)
1	L-lactate dehydrogenase OS=*Lactobacillus helveticus*	174	35,089
2	Enolase OS=*Streptococcus suis* (strain 98HAH33)	234	47,066
3	60 kDa chaperonin OS=*Lactobacillus brevis* (strain ATCC^®^ 367 / JCM 1170)	92	57,044
4	Phosphoenolpyruvate-protein phosphotransferase OS=*Lactococcus lactis subsp. lactis* (strain IL1403)	137	62,521

MOWSE, molecular weight search.

MW, molecular weight; kDa, kilodalton.

### Detection of putative virulence factor genes by polymerase chain reaction

**FIGURE 5:**
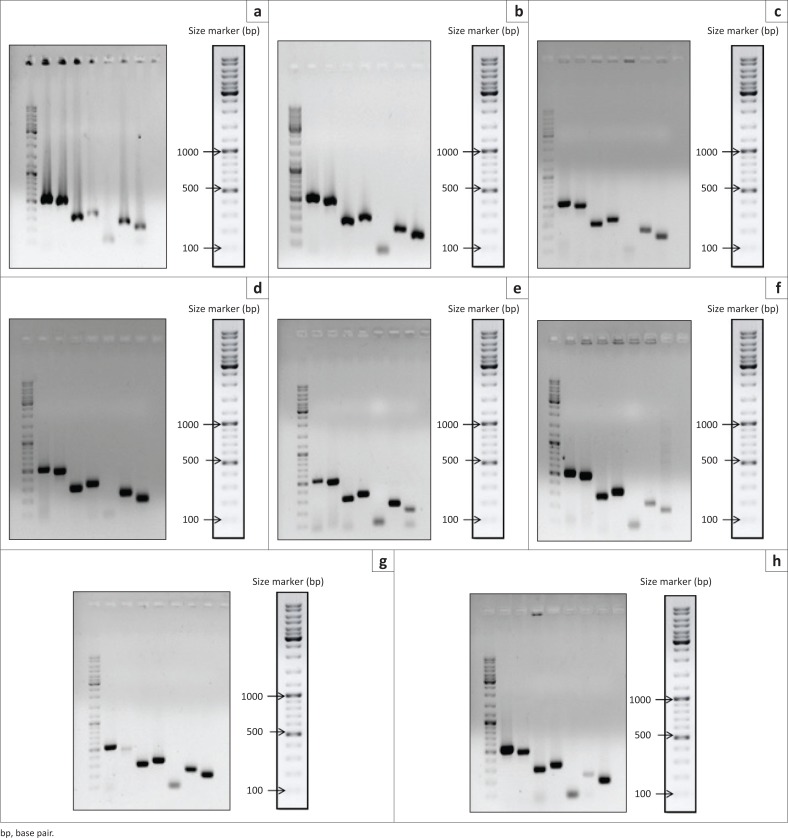
Putative virulence factor genes were detected by polymerase chain reaction assays. Products are shown visualised on 1% agarose gels. The first lanes on all gels are size markers, and the subsequent lanes represent virulence genes in the following order: *hly1*; *hly2*; *hly3*; *nox*; *sod*; *pavA*; *psaA*. (a), A1; (b), A2; (c), A3, (d), A5; (e), A6; (f), A11; (g), A12; (h), NCFB657.

Visualisation of PCR products by gel electrophoresis, as presented in Figure 5, followed by sequencing and nucleotide database search confirmed the presence of seven candidate virulence genes in genomes of seven South African isolates as well as an avirulent reference strain, NCFB657.

## Discussion

All putative virulence genes under investigation were present in the genomes of all South African isolates used in this study, as well as the reference strain NCFB657. These results are in accordance with the findings of Türe and Altinok (2016), who reported the presence of this set of putative virulence factor genes in the genomes of a total of 34 *L. garvieae* strains, including the avirulent reference strains ATCC^®^ 49156 and ATCC^®^ 4392 and isolates from diseased rainbow trout in Turkey, France, Iran, Spain and Italy.

No phenotypic proof of the presence of a polysaccharide capsule was collected in this study using light microscopy. These observations alone, however, should not be considered definitive proof of the absence of capsules, as Yoshida et al. (1997) reported that neither negative staining using Muir’s method or Indian ink, nor the Quellung reaction was successful in visualising *L. garvieae* KG^−^ capsules.

The EPS capsule influences a variety of host interactions, including adhesion, invasion and sensitivity to opsonophagocytosis, and is a well-recognised virulence factor of *L. garvieae.* The *eps* locus of *L. garvieae* Lg2 was reported to be a genomic island, attributed to the presence of ISs IS982 on both ends of the EPS biosynthesis cluster, differences in guanine-cytosine (GC) content between the Lg2 chromosomal average and cluster (39% and 31%, respectively) and demonstration that this locus was probably inserted into the locus syntenic to four sequenced *L. lactis* strains (Morita et al. 2011). It was postulated that this gene cluster might have been present in the avirulent non-capsulated reference strain ATCC^®^ 49156, originally isolated from diseased yellowtail, and was lost during repeated subculturing (Morita et al. 2011). The negative results obtained using the PCR amplification of the EPS biosynthesis cluster in this study may reflect a similar phenomenon as the attenuation of ATCC^®^ 49156 by repeated subculturing. The strains used in this study were isolated from diseased rainbow trout in 1986 and has been repeatedly subcultured under laboratory conditions, which may be selective for the non-capsulated phenotype as no selective pressure in the form of host immune mechanisms is applied. Presence of the capsule confers fitness only when growth occurs under *in vivo* conditions. Therefore, deletion of the EPS biosynthesis cluster, and subsequently the loss capsulated KG^−^ phenotype, is a possible explanation for the results obtained. However, in light of recent findings by Türe and Altinok (2016), the isolates investigated in this study may not have been capsulated at the time of isolation.

Previous immunoproteomic studies on *L. garvieae* did not identify enolase, 60 kDa chaperonin or phosphoenolpyruvate-protein phosphotransferase as antigens recognised by rabbit antisera (Shin et al. 2007) or olive flounder (*Paralichthys olivaceus*) antisera (Shin et al. 2009). However, L-lactate dehydrogenase was reported as an antigen common to both KG^+^ and KG^−^ serotypes (Shin et al. 2009). Although enolase, 60 kDa chaperonin and phosphoenolpyruvate-protein phosphotransferase have not been identified as antigens in *L. garvieae*, the identification of these extracellular proteins is of interest as they have been described as moonlighting proteins with involvement in virulence and regulation of virulence genes in Gram-positive bacteria.

Enolase (EC 4.2.1.11) is a glycolytic enzyme responsible for catalysing the conversion of 2-phosphoglycerate to phosphoenolpyruvate in the second last step of glycolysis, and the reverse reaction in gluconeogenesis (Lee et al. 2006). Additionally, this enzyme plays a role in virulence of Gram-positive and Gram-negative bacteria (Bergmann et al. 2001; Nogueira et al. 2013; Pancholi & Fischetti 1998). Numerous studies on the moonlighting activities of enolase in prokaryotes have highlighted its involvement in binding of host plasmin(ogen), a process of critical importance to invasion of host tissues (Bergmann et al. 2001; Henderson & Martin 2011; Pancholi & Fischetti 1998). Binding of host plasmin(ogen) to its surface endows the bacterial cell with protease activity, which facilitates invasion and dissemination in the host (Winram & Lottenberg 1996). Surface-associated enolase with plasmin(ogen) binding activity has been reported in human streptococcal pathogens *Streptococcus pyogenes* (Pancholi & Fischetti 1998), *Streptococcus pneumoniae* (Bergmann et al. 2001) and piscine pathogen *Streptococcus iniae* (Kim et al. 2007). A study by Wang et al. (2015) confirmed the immunogenicity of *S. iniae* recombinant α-enolase in mice, showing that immunisation with α-enolase elicited a significant increase in specific immunoglobulin G in comparison with the control group and ultimately protected mice against systemic *S. iniae* infection (Wang et al. 2015). The ability of *S. iniae* α-enolase to confer protection in Nile tilapia (LaFrentz, Shoemaker & Klesius 2011) and turbot (*Scophthalmus maximus*) (Zhang, Zhang & Sun 2014) has been previously suggested.

The prokaryotic 60 kDa chaperonins (Cpn60) is a highly conserved group of proteins that mediate intracellular protein folding to ensure correct functioning (Henderson, Fares & Lund 2013). Cpn60 is found on the cell surface of a variety of bacteria where it functions mainly as an adhesin. Among the members of the order Lactobacillales reported to produce extracellular Cpn60 are *Lactobacillus johnsonii* (Bergonzelli et al. 2006), *L. lactis* (Katakura et al. 2010), *Streptococcus agalactiae* (Hughes et al. 2002) and *Streptococcus suis* (Wu, Zhang & Lu 2008). Diverse ligands of bacterial cell-surface Cpn60 have been identified, ranging from mucin, lactoferrin, invertase and glycosphingolipids to integrin receptors CD11/CD18, *α*_v_*β*_3_ and dendritic cell-specific intercellular adhesion molecule-3-grabbing non-integrin (Henderson et al. 2013). Proteomic analysis of *S. iniae* ATCC^®^ 29178 and *L. garvieae* KG9408 (capsulated) by 2-dimensional gel electrophoresis identified Cpn60 in both fish pathogens (Shin et al. 2006), but subsequent immunoproteomic analyses by immunoblotting with rabbit and olive flounder antiserum did not identify this protein as an antigen of either capsulated or non-capsulated *L. garvieae* (Shin et al. 2007, 2009).

Phosphoenolpyruvate (PEP)-protein phosphotransferase (EC 2.7.3.9) is a key enzyme in the bacterial phosphotransferase system (PTS) (Deutscher et al. 2014). The multifunctionality of *S. pneumoniae* PEP-protein phosphotransferase (PtsA) has been investigated by demonstrating its presence on the cell surface of *S. pneumoniae* using immunofluorescence techniques and using recombinantly expressed PtsA (rPtsA) to screen a filamentous phage display library to identify peptides capable of inhibiting adhesion of *S. pneumoniae* to human lung adenocarcinoma cells (Mizrachi Nebenzahl et al. 2016). These peptides showed homology to various human ECM proteins, including multimerin I, protocadherin 19 and collagen type VIIα1, suggesting that PtsA acts as an adhesin of *S. pneumoniae* (Mizrachi Nebenzahl et al. 2016). Furthermore, rPtsA was identified as a candidate target for vaccine development by showing that immunisation of mice with rPtsA offered protection against pneumococcal challenge via different routes (Mizrachi Nebenzahl et al. 2016).

Pore-forming toxins are secreted by a variety of Gram-negative and Gram-positive pathogenic bacteria to disrupt lipid bilayers of host cells (Iacovache, van der Goot & Pernot 2008). The putative virulence gene *hly1* was reported to display 56% amino acid sequence homology to a protein in *E. faecalis* containing motif PF03006 conserved in proteins with haemolytic activity (Morita et al. 2011). Proteins containing this motif are classified in the haemolysin-III related family (http://pfam.xfam.org/; Finn et al. 2014). Based on the structural features of *hly*1, it is classified as a member of the α-pore-forming toxins, which include colicins of *Escherichia coli* (Iacovache et al. 2008). Another putative haemolysin under investigation in this study, encoded by *hly2*, was reported to display 72% amino acid homology to with a haemolysin in *S. suis* (Morita et al. 2011). The gene product of *hly3* was reported to display 59% amino acid sequence similarity to a haemolysin in *S. pyogenes* (Morita et al. 2011), an organism associated with *β*-haemolytic activity.

Reactive oxygen species (ROS) have various noxious effects on cells (Yesilkaya et al. 2000). Protection against oxidative stress is afforded by the enzymatic removal of ROS by catalase and SOD. The LAB are catalase negative (Collins et al. 1983); therefore, SOD (EC 1.15.1.1) represents a major defence mechanism against oxidative stress in *L. garvieae*. The detoxifying properties of SOD can also enhance intraphagocytic survival of bacteria upon generation of bactericidal ROS as a result of respiratory burst (Fang et al. 2015). The enzyme NOX reduces molecular oxygen to H_2_O or H_2_O_2_ and was proposed to perform an important role in defence against O_2_ toxicity and O_2_ sensing, which are essential functions during aerobic growth of LAB (Muchnik et al. 2013). The importance of NOX in the virulence of *S. pneumoniae* has been established by Yu et al. (2001) by demonstrating significant attenuation of a *nox*-deficient mutant, leading to the conclusion that NOX is required for *in vivo* proliferation in O_2_-rich environments. Random peptide library phage display was used to identify several extracellular matrix proteins as ligands to recombinant NOX, suggesting that the enzyme may play a multifunctional role in virulence by additionally acting as an adhesin (Muchnik et al. 2013).

Adherence to host cells is a prerequisite step in the colonisation processes of pathogenic and commensal microbes (Kline et al. 2009). Morita et al. (2011) reported that protein LCGL_1330 of *L. garvieae* Lg2 shared 62% amino acid identity with *S. pneumoniae* TIGR4 PavA and contained a fibronectin-binding motif (PF05833). Pneumococcal adherence and virulence factor A (PavA) is a cell surface-localised fibronectin-binding protein that lacks both typical signal sequences for secretion as well as the LPXTG anchorage motif characteristic of cell surface proteins (Holmes et al. 2001). Another protein with amino acid sequence homology (49%) to a *S. pneumoniae* cell surface-exposed virulence determinant, pneumococcal surface antigen A (PsaA), was identified in Lg2 by Morita et al. (2011). This protein was originally identified as a putative adhesin (Berry & Paton 1996); however, its structure was not found to be consistent with the function of adhesion (Johnston et al. 2004). Its function was revealed to be a divalent metal ion-binding lipoprotein component of an ATP-binding cassette (ABC) transporter for Mn^2+^ (Johnston et al. 2004).

## Conclusion

The major virulence factor of *L. garvieae* defined thus far is the antiphagocytic polysaccharide capsule. With the aid of next-generation sequencing of full genomes, several other putative virulence factors have been identified. Despite rapid advances in the areas of molecular biology and bioinformatics, its pathogenic processes are inadequately understood. No polysaccharide capsule was detected in any of the South African strains studied. Putative virulence factors with roles in adhesion, cytolytic activity, oxidative stress tolerance and metal homeostasis were identified in a set of seven South African fish pathogenic *L. garvieae* isolates, as well as an avirulent isolate. Detection of extracellular proteins of South African fish pathogenic *L. garvieae* isolates in this study identified candidates that may be investigated as subunit vaccines in future.

Specific virulence factors responsible for the pathogenicity of *L. garvieae* could not be identified, as putative virulence factor genes were present in both the fish pathogenic isolates and the avirulent isolate. All virulence factors discussed in this study, with the exception of haemolysins, are virulence lifestyle factors that indirectly contribute to host damage. These virulence lifestyle factors aid in the infection process by evasion of the host’s innate immune system, cofactor homeostasis, systemic invasion and dissemination in the host and adhesion to host tissues. Future studies should focus on investigating differential expression of virulence lifestyle and true virulence genes during growth in the host environment versus laboratory conditions.
